# Establishment of a virtual transborder tumor board for cancer patients in Central and Southeastern Europe: An initiative of the Central European Cooperative Oncology Group (CECOG)

**DOI:** 10.1007/s00508-022-02016-z

**Published:** 2022-03-21

**Authors:** Christiane Thallinger, Peter Berzinec, Emina Bicakcic, Adelina Dan, Gabriella Fabian, Laurentia Nicoletta Gales, Cvetka Grasic Kuhar, Urska Janzic, Zsusanna Kahan, Marina Mencinger, George Penthedourakis, Joseph Sgouros, Luka Simetic, Daniela Sirbu, Milan Vosmik, Anna Wrona, Christoph Zielinski

**Affiliations:** 1 Central European Cooperative Oncology Group, Ohmanngasse 26, 1190 Vienna, Austria; 2 Department of Medicine I, Medical University Vienna—General Hospital, Waehringer Guertel 18–20, 1090 Vienna, Austria; 3 Department of Oncology at the Hospital of St Zoerardus Zobor, Nitra, Teaching Base of the Slovak Medical University Bratislava, Bratislava, Slovakia; 4 Oncology Unit, Clinical Center of Sarajevo University, Sarajevo, Bosnia and Herzegovina; 5 Department of Medical Oncology at Ion Chiricuta Institute of Oncology, Cluj-Napoca, Romania; 6 Department of Oncotherapy, University of Szeged, Szeged, Hungary; 7 Department of Oncology at UMF “Carol Davila”, Bucharest, Romania; 8 Department of Medical Oncology, Institute of Oncology Ljubljana, Ljubljana, Slovenia; 9 Department of Medical Oncology, University Clinic Golnik, Golnik, Slovenia; 10 Department of Medical Oncology, University Hospital of Ioannina, Ioannina, Greece; 11 3rd Medical Oncology Department, “Agii Anargiri” General Hospital and Cancer Center, Athens, Greece; 12 Department of Oncology, University Hospital Centre Zagreb, Zagreb, Croatia; 13 Oncohelp Oncology Center, Timisoara, Romania; 14 Department of Oncology and Radiotherapy, University Hospital Hradec Králové, Hradec Králové, Czech Republic; 15 Department of Oncology and Radiotherapy, Medical University Gdansk, Gdansk, Poland; 16 Vienna Cancer Center, Vienna Hospital Association and Medical University Vienna, Vienna, Austria

**Keywords:** Central and Southeastern Europe, Remote tumor board, Immune checkpoint inhibitors, Malignant diseases, CECOG

## Abstract

**Purpose:**

To establish a transborder virtual tumor board (VTB) fostering state-of-the-art management of cancer patients by exchanging knowledge and expertise among oncologists in Central and Southeastern Europe (CEE).

**Methods:**

We established and implemented a VTB based on the WebEx platform. This allowed for password-protected and secure upload of patient cases to be presented and discussed among colleagues from various oncology centers scattered throughout CEE in order to arrive at a recommendation for further diagnoses and/or treatment.

**Results:**

A total of 73 cases from 16 oncology centers located in 11 CEE countries were uploaded by 22 physicians; 71 were discussed over the course of 17 virtual meetings between June 2018 and May 2019 and 12 different kinds of malignant diseases were discussed with lung cancer (46.6%), melanoma (19.2%) and bladder cancer (13.6%) being the most commonly presented tumor entities. Of the discussed patients, 93.3% had stage IV disease at the time of presentation, 62.6% received chemotherapy or targeted treatment and 67.1% were treated with immune checkpoint inhibitors (ICPIs). The most common causes for presentation and discussion of patient cases were related to the use of ICPIs (80%).

**Conclusion:**

When the need for expertise exceeds locally available resources, web-based VTBs provide a feasible way to discuss patient cases and arrive at conclusions regarding diagnoses and/or treatment across large geographic distances. Moreover, VTBs provide an innovative way for proper, state-of-the-art management of patients with malignant diseases in times of social distancing and the resulting need for restricted interaction during the current SARS-CoV‑2 (severe acute respiratory syndrome coronavirus type 2) pandemic.

## Introduction

The Central European Cooperative Oncology Group (CECOG) has been active in the area of Central and Southeastern Europe (CEE) since 1999. The CECOG has conducted controlled clinical trials, provided continuing medical education and recently also provided analyses regarding access to drugs in an area which is very often characterized by protracted reimbursement decisions on European Medicines Agency (EMA)-registered medicines [1, 2].

**Fig. 1:**
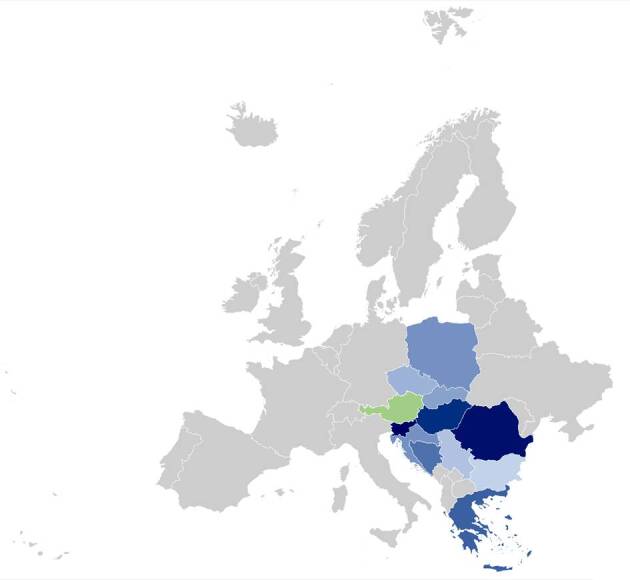
Participating countries in Central and Southeastern Europe. *Shades of blue* denote the number of presented cases with darker hues representing more cases (see Table 1); *green* represents the location of the CECOG Headquarters in Austria. Participating CEE countries: Bosnia and Herzegovina, Bulgaria, Croatia, Czech Republic, Greece, Hungary, Poland, Romania, Serbia, Slovak Republic, Slovenia. *CECOG* Central European Cooperative Oncology Group, *CEE* Central and Southeastern Europe

The CEE is a large geographic area (Fig. 1) where access to EMA-registered drugs is hampered by varying national reimbursement strategies. Moreover, quality-oriented day to day decisions in individual cancer care in certain geographic areas may exceed available resources [3].

Access to high-quality care in an age of precision medicine and immune checkpoint directed therapeutic interventions with ever growing numbers of indications have become critical in providing state-of-the-art treatment according to not only registrations by the EMA, but also ongoing clinical research. The latter two aspects are of special interest in regions with the constraints of limited resources where decisions on reimbursement are made on national levels. These circumstances pose limitations in clinical experience in drug handling, particularly of recently approved and registered drugs which have added enormously to the treatment armamentarium of malignancies, such as immune checkpoint inhibitors (ICPIs), yet pose a challenge regarding their use, the duration of treatment, decision-making in the case of mixed responses as well as toxicity and its handling.

All the above considerations let to the decision of CECOG to set up a multidiscipilnary, transborder virtual tumor board (VTB) in CEE. This VTB would offer an opportunity to connect oncologists and cancer centers in the form of a virtual network in order to enable appropriate guideline-driven multidisciplinary evaluation and cancer care [4–8] with the main emphasis upon training in the use of ICPIs in order to facilitate access to this important group of newly developed drugs in the region.

The present report describes the methodology used to establish a transborder VTB and its feasibility regarding presented patients, their diagnoses and treatment recommendations in an area of frequently limited financial resources. We believe that such a tool could be used to facilitate and enhance cancer care in other parts of the world with similar or comparable circumstances. Needless to say, the intercurrent SARS-CoV‑2 pandemic would further support the virtual approach.

## Methods

### Establishment of a transborder VTB

An electronic platform based on the WebEx (Cisco Systems, Inc., San Jose, CA, USA) teleconference system has been established with the help of create-mediadesign GmbH, Vienna, Austria. The platform enabled the password-protected upload of patient histories. The platform was provided with the ability to be joined by clinicians from the area of CEE selected from 150 academic centers in 23 countries partnering with CECOG by virtue of their high expertise in the field. From the very start and according to the topic of the competitively acquired grant, it was made clear that the content of the platform would serve discussions regarding indications and complications of CPI treatment in various malignancies. Initially, a group of 12 selected investigators consisting of chairpersons of oncology departments of high reputation from the area with balanced distribution among various countries were invited to the CECOG headquarters in Vienna, Austria, for setting up the tumor board procedures in February 2018. At this point, the VTB was first presented to a larger audience. These persons were asked to nominate important hospitals with appropriate points of gravitation in the field from each individual country resulting in 22 clinicians from 16 centers in 11 countries in the CEE area. A training session on access to the platform, upload of data and technical aspects of clinicians was subsequently provided to the involved centers.

### Implementation of the transborder VTB

At the initial meeting, it was agreed that the large number of participating centers would preclude weekly conferences among all participants. Thus, biweekly meetings with changing participants were agreed. The first tumor board was held in June 2018 and the concept was followed for 1 year during which 73 cases were uploaded to the system and 71 cases were actively discussed; all 73 uploaded cases were evaluated for the purpose of the present report.

Centers and clinicians scheduled for participation were contacted by the CECOG head-office 5 days in advance and asked to upload their cases including patient histories and radiological evaluations.

Each VTB was moderated by the CECOG head office, and a protocol of each tumor board was prepared and uploaded to the platform. Tumor boards were chaired by one of the involved clinicians (C.Z., T.Cu., T.Ci.).

### Evaluation of the transborder VTB

Success of the VTB was evaluated using knowledge questionnaires before and after an initial training session on ICPI treatment (spring 2018) and again at the end of the VTB period (spring 2019). A survey was sent to the participants in July 2021 to evaluate the value of the VTB to the participants. The supplement shows all questionnaires and the respective results.

### Ethics

All patient data were presented to the VTB anonymized at the level of month/year of birth, ethnicity and biological sex. Patient consent for aggregated evaluation of their cases within the present project was obtained.

Ethics committee approval for conducting these VTB meetings was not required as per local law.

## Results

### Included cases per country and per site

A total number of 73 cases from 16 oncology centers located in 11 countries in the CEE region were uploaded to the VTB system by 22 physicians and 71 were presented and discussed over the course of 17 virtual meetings between June 11th, 2018 and May 27th, 2019; all 73 uploaded cases are discussed here. On average 4.6 cases from each center and 3.3 cases per physician were presented and discussed (Table 1).

**Table 1:** Participating countries and distribution of centers, physicians and cases

Countries *N* = 11	Centers *N* = 16	Physicians *N* = 22	Cases *N* = 73
Slovenia	2	4	14
Romania	3	4	13
Hungary	1	3	11
Greece	2	3	8
Bosnia and Herzegovina	1	1	7
Poland	1	1	5
Croatia	1	1	5
Slovakia	1	1	4
Czech Republic	1	1	3
Serbia	2	2	2
Bulgaria	1	1	1
*Mean*	*1.5 centers per country*	*2.0 physicians per country*	*6.6 cases per country*
	*–*	*1.4 physicians per center*	*4.6 cases per center*
	*–*	*–*	*1.5 cases per physician*

Different types of malignant diseases were recorded (Table 2). Lung cancer (46.6%), melanoma (19.2%) and bladder cancer (13.6%) were the most commonly presented tumor entities and accounted for 80% of all cases. The majority of discussed patient cases had advanced stages of disease, 93.3% had evidence of metastasis at time of presentation, 62.6% had previously received chemotherapy or targeted therapy, 60.3% had undergone surgery, 49.3% had received radiotherapy, 20 patients (27.4%) had undergone all 3 different treatment modalities and 50 patients (67.1%) received ICPI treatment.

**Table 2:** Clinical characteristics of patients presented at the VTBs

Tumor entity	Presented cases		Presence of metastases		Prior surgery		Prior radiotherapy		Prior chemotherapy or targeted therapy		Prior or ongoing ICPI therapy	
	*n*	%	*n*	%	*n*	(%)	*n*	(%)	*n*	(%)	*n*	(%)
Lung cancer	34	467	33	97	11	32	18	53	27	79	27	79
Melanoma	14	19	13	93	13	93	4	29	6	43	11	79
Bladder cancer	10	14	9	90	9	90	5	50	8	80	5	50
Renal cell cancer	5	7	5	100	5	100	2	40	4	80	2	40
Colorectal cancer	2	3	2	100	1	50	1	50	2	100	1	50
Laryngeal cancer	2	3	2	100	1	50	2	100	2	100	0	0
Anal carcinoma	1	1	1	100	0	0	1	100	1	100	1	100
Merkel cell carcinoma	1	1	1	100	1	100	0	0	0	0	1	100
Mesopharyngeal cancer	1	1	0	0	1	100	1	100	1	100	0	0
Salivary ductal carcinoma	1	1	1	100	1	100	1	100	1	100	0	0
Uveal melanoma	1	1	1	100	1	100	1	100	1	100	1	100
CUP	1	1	–	–	–	–	–	–	–	–	–	–
*Total*	*73*	*100*	*68*	*93*	*44*	*60*	*36*	*49*	*53*	*63*	*49*	*67*

*CUP* carcinoma of unknown primary, *ICPI* immune checkpoint inhibitor, *VTB* virtual tumor board

### Educational value of the VTB

**Fig. 2:**
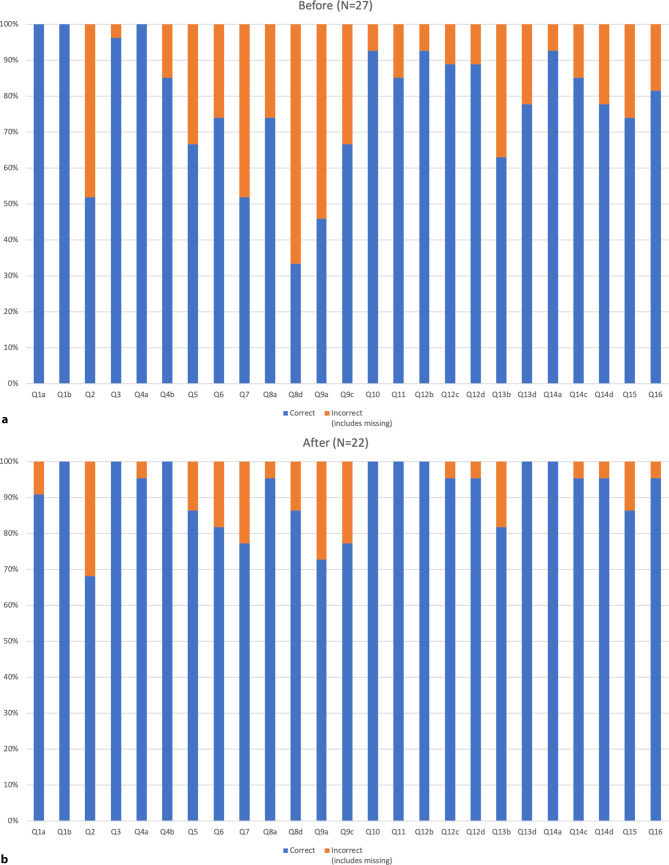
Evaluation of the educational effect of the initial ICPI training. Panel **a**. Before educational training session. Panel **b**. After educational training session. The list of questions is provided in the online supplemental material

**Fig. 3:**
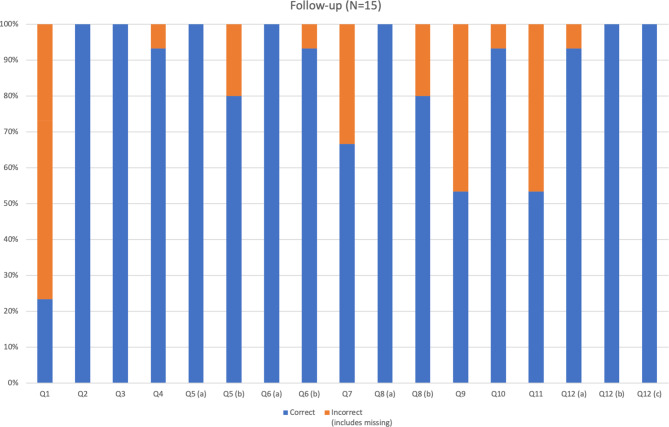
Evaluation of the educational effect of the ICPI training at re-evaluation after 1 year. The list of questions is provided in the online supplemental material

The results of the questionnaires provided before and after the initial training session showed a clear improvement in the percentage of correct responses, with the number of questions answered correctly by fewer than 80% of the respondents decreasing from 12 to 4 (Fig. 2a,b). The follow-up evaluation conducted after approximately 1 year contained a different set of questions with the majority of questions being answered correctly by 80% or more of participants (Fig. 3). The corresponding questions and correct answers can be found in the online supplementary material.

### Value of the VTBs to participants

The post-VTB survey was completed by 14 participants from 11 countries. Of 14 respondents, 8 (57%) rated the quality of the VTB meetings as excellent, 5 (36%) as good, 1 (7%) as average. Personal motivations to participate were continuous medical education (*n* = 6, 43%), specific questions on particular cases (*n* = 6, 43%), and networking (*n* = 2, 14%); these motivations were fulfilled in everybody’s view. Especially when their experience with ICPIs was lower, the VTB helped to increase knowledge.

**Fig. 4:**
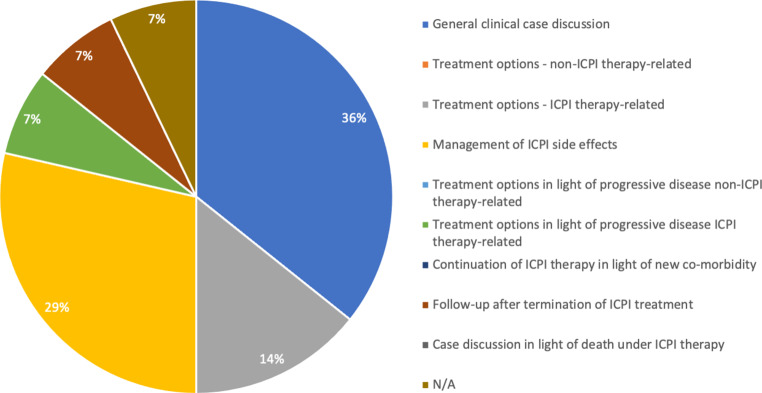
Main motivation for presenting a particular case. In the case of more than one presented case, participants were asked to state the motivation of presenting the case most important to them

The main motivation to present a particular case was a general clinical case discussion (*n* = 5, 36%), management of ICPI side effects (*n* = 4, 29%), and the discussion of ICPI-related treatment options (*n* = 2, 14%; Fig. 4). Participants’ expectations with regards to their main motivation were met in 86% of cases (*n* = 12); one respondent each stated no or not applicable. Learning from their own or other participants’ cases related mostly to ICPI side effect management, treatment sequencing and decision making after progression from ICPIs, reasons for deterioration of the disease, and getting confirmation from others of the treatment decision taken.

For future educational sessions, participants suggested to learn on clinical trial initiatives and collaboration with other specialties, treatment sequencing, ICPI side effect management, ICPIs in combination with other treatments, new compounds and other tumor board formats, such as molecular tumor boards.

## Discussion

The presented period of TIGER virtual tumor boards was conducted biweekly over 1 year in countries of central and southeastern Europe. At the time participants had little to no personal experience with ICPI therapy. Accordingly, physicians participated for reasons of continuous medical education or because they had specific questions to the VTB, largely around general case discussions and if ICPIs were an option in their presented cases and side effect management. These individual expectations were met in the vast majority of cases.

Tumor boards drawing on the expertise of multidisciplinary teams have become standard worldwide as an approach to discuss complex patient cases and arrive at well-founded treatment decisions in the management of cancer patients. The CECOG was founded in Vienna, Austria, in the year 1999 and established a number of committees with the objective to harmonize among western, central and southeastern European countries the implementation of treatment standards resulting from medical and scientific research in the treatment of cancer patients.

This initiative rests on three pillars: (1) multi-country clinical trials, (2) the CECOG Academy, and (3) a professional society involved in improving cancer care through the support of local reimbursement negotiations, patient advocacy, patient registries, etc. The initial goals of the TIGER VTBs were to improve the understanding of the immune system and its role in cancer. Further we wanted to inform on outcomes of ICPI treatments in various indications and on options of combination therapies to ameliorate treatment outcomes with currently available clinical examples and an outlook on upcoming options, to educate on the use of ICPIs in special clinical situations and to guide participants regarding the management of side effects (https://academy.cecog.org/about-tiger/).

Transborder mentoring initiatives, such as this one, are especially important for clinicians from low to middle-income countries who often do not have experienced colleagues from multiple disciplines in house. Also, they act as networking incubators, especially for the rising generation, and can offer access to clinical trials. An initiative from Latin America followed a similar approach with a web-based pediatric brain tumor board involving clinicians from 15 countries [9]. The most frequently asked questions to the Latin American Tumor Board (LATB) also revolved around questions regarding treatment, second opinions, routine case presentations and alternative diagnostic or therapeutic options. Uncertainty about the best therapeutic approach was the main motivation for the case presentation to experienced colleagues. When treatment options are generally available but difficult to access in a certain region, the personal experience and the respective learning curve are often lagging behind more affluent regions.

Our survey conducted approximately 2 years after the end of the presented period of the VTB clearly shows that this initiative helped when clinicians’ experience was low and increased their knowledge, especially with regard to the management of side effects of the new class of drugs. i.e. ICPIs and the understanding of unexpected disease development.

It is a limitation of the present project that we did not systematically evaluate the recommendations made to the participants, if the VTB confirmed the presented approach or recommended alternative treatments, additional diagnostic testing etc., and if the recommendations were followed and with what outcome. Only few cases (*n* = 12) were presented a second time for follow-up discussion.

Others have investigated the impact of multidisciplinary tumor boards on the quality of diagnosis and treatment and found that between 4% and 45% of presented cases saw changes in the diagnosis or staging and between 4.5% and 52% recommendations saw changes in the management plan, as summarized by Habermann et al. [10]. The systematic analysis of the feasibility of the recommendations made at the abovementioned LATB showed that only 64% of all recommendations and 60% of diagnostics-related recommendations could be implemented [9], while on-site tumor boards were reported to result in 75–90% of feasible recommendations [11, 12].

Indeed, our survey showed that cost and reimbursement were important obstacles hindering the implementation of the recommendations made. Thus, professional societies and advocacy groups will have to continue in their efforts to forge compromises between payors and the pharmaceutical industry to allow for rapid reimbursement of important new medicines also in low and middle-income countries.

In the current pandemic times, we have learned to embrace virtual means of personal and educational exchange and the available tools have become more user-friendly. Initiatives such as the TIGER VTBs will increase in number and will enhance the interconnectedness of clinicians regionally and worldwide. This knowledge exchange will support clinicians with limited possibilities for personal experience with certain new drugs or diagnostic procedures to swiftly learn from each other and from experienced colleagues. Future VTBs should systematically evaluate the feasibility of recommendations made by the group of experts in order to improve patient outcomes and to identify and facilitate new research initiatives.

## Abbreviations

CECOG: Central European Cooperative Oncology Group
CEE: Central and Southeastern Europe
EMA: European Medicines Agency
ICPIs: Immune checkpoint inhibitors
NSCLC: Non-small cell lung cancer
VTB: Virtual tumor board
